# Dominance of Attention Focus and Its Electroencephalogram Activity in Standing Postural Control in Healthy Young Adults

**DOI:** 10.3390/brainsci12050538

**Published:** 2022-04-22

**Authors:** Shun Sawai, Shoya Fujikawa, Shin Murata, Teppei Abiko, Hideki Nakano

**Affiliations:** 1 Graduate School of Health Sciences, Kyoto Tachibana University, Kyoto 607-8175, Japan; sawai.neuroreha@gmail.com (S.S.); murata-s@tachibana-u.ac.jp (S.M.); abiko@tachibana-u.ac.jp (T.A.); 2 Department of Rehabilitation, Kyoto Kuno Hospital, Kyoto 605-0981, Japan; 3 Department of Physical Therapy, Faculty of Health Sciences, Kyoto Tachibana University, Kyoto 607-8175, Japan; a903019130@st.tachibana-u.ac.jp

**Keywords:** attention focus, dominance, postural control, motor performance, EEG

## Abstract

Attention focus changes performance, and external focus (EF) improves performance compared to internal focus (IF). However, recently, the dominance of attention focus, rather than the effectiveness of unilateral EF, has been examined. Although the positive effects of EF on standing postural control have been reported, the dominance of attention focus has not yet been examined. Therefore, the purpose of this study was to examine the dominance of attention focus and its neural mechanism in standing postural control using electroencephalography (EEG). A standing postural control task under IF and EF conditions was performed on healthy young men. Gravity center sway and cortical activity simultaneously using a stabilometer and an EEG were measured. Participants were classified into IF-dominant and EF-dominant groups according to their index of postural stability. The EEG was analyzed, and cortical activity in the theta-wave band was compared between the IF-dominant and EF-dominant groups. Significant neural activity was observed in the left parietal lobe of the IF-dominant group in the IF condition, and in the left frontal lobe of the EF-dominant group in the EF condition (*p* < 0.05). Differences in EEG activity between IF-dominant and EF-dominant groups, in standing postural control, were detected. This contributes to the development of training methods that consider attentional focus dominance in postural control.

## 1. Introduction

Attention focus changes performance. There are two types of attention focus: internal focus (IF) and external focus (EF), where IF directs attention to the inside of the body, and EF directs attention to the outside of the body [1]. Previous research [2] reported that performance was improved by focusing attention on EF compared to IF. As examples, the effectiveness of EF has been tested in movements that require accuracy, such as dart shooting [3,4] and golf putting [5,6]. The effects of attentional focus on EF are explained by the constrained action hypothesis [7], in which the conscious control of movements inhibits the automatic system and constrains movement. In EF, inhibiting the conscious control of movements automates movement and improves performance. By contrast, Castaneda et al. [8] and Perkins-Ceccato et al. [9] reported that low-ability participants performed better in IF than in EF where low-ability participants paid more attention to each step of the movement when they performed it accurately. Hence, performance in the IF condition, in which attention is directed to movement, may improve in low-ability participants.

Attention focus also influences standing postural control. Many previous studies have examined the effects of attention focus in healthy young adults [10,11] and healthy elderly adults [12,13] and reported improvements in postural control in EF compared to IF. The effects of attention focus occur not only in healthy individuals, but also in those with diseases. EF has been reported to be effective in controlling the standing posture in patients with Parkinson’s disease [14,15], stroke patients [16], and after ankle sprain [17,18]. Therefore, EF has been shown to improve standing postural control [2]. Sherman et al. [19] conducted a basic study using an electroencephalogram (EEG) to reveal the neural basis of attention focus and reported that frontal lobe theta power increased in EF compared with IF. Thus, brain function was related to performance by focusing on the IF and EF.

Sakurada et al. [20] reported that, regardless of the ability level, there were two groups that performed better with attention focus on IF (IF-dominant group) and with attention focus on EF (EF-dominant group) in an upper limb tracking task. Sakurada et al. [21] conducted a basic study using functional near-infrared spectroscopy in the IF-dominant and EF-dominant groups and observed that the activity of the right dorsolateral prefrontal cortex and the right somatosensory association cortex was lower when the task was performed with optimal attentional focus than when it was not. Furthermore, Sakurada et al. [22] used EEG to analyze event-related potentials and showed that somatosensory and visual information processing differed between the IF-dominant and EF-dominant groups. Thus, optimal attentional focus was dominant, and differences existed in cortical activity.

However, the dominance of attention focus in standing postural control and its neural basis have not been sufficiently investigated. Attention focus was accompanied by cognitive load [10], and cognitive load has been shown to increase frontal lobe activity [23]. In addition, attention focus required attentional functions and attention was associated with frontal to parietal lobe activity [24]. Therefore, in this study, characteristic EEG of the frontal and parietal lobes related to cognition and attention were expected. Revealing this may aid in providing a training method for improving standing balance that adapts to an individuals’ optimal attention focus. Hence, this study aimed to reveal the dominance of attention focus during standing postural control and to examine the neural basis using EEG.

## 2. Materials and Methods

### 2.1. Participants

Thirty-one healthy young men (21.1 ± 0.73 years) were recruited for this study. Gender differences in standing postural control have been previously reported and brain structure and cognitive aspects were related [25]. In addition, gender differences in attentional function [26] and the effects of attention focus have also been shown [27]. For these reasons, only healthy young men were included in this study in order to unify the participant characteristics. All participants were confirmed to have no history of disease presenting with motor or cognitive impairments and had normal or corrected-to-normal vision. This study was conducted in accordance with the Declaration of Helsinki, and informed consent was obtained from all the participants. This study was approved by the local institutional ethics committee of Kyoto Tachibana University.

### 2.2. Study Protocol

**Figure 1:**
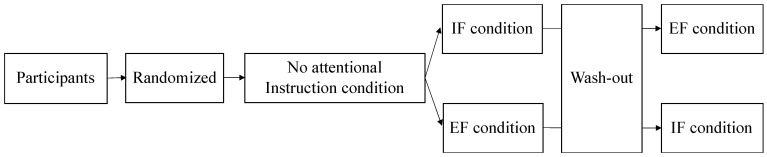
At first, the participants performed the task in the “no attentional instruction” condition without attentional focus. The participants were randomly divided into two groups. One group performed the IF condition followed by the EF condition, and the other group performed the EF condition followed by the IF condition. The participants performed a washout task with no attention focus in between the attention-focused tasks. Attention was focused on the inside and outside of the body in the IF and EF conditions, respectively. IF: internal focus; EF: external focus.

This study was a randomized crossover design based on a previous study [20]. All participants first performed a postural control task under the “no attentional instruction” condition. Participants were then randomly divided into two groups. One group performed the task in the IF condition and then performed the task in the EF condition. The other group performed the task in the EF condition, followed by the task in the IF condition. Between the attention-focusing tasks, the “no attentional instruction” condition was performed as a washout task (Figure 1). In addition, a 1 min break was included between each task.

**Figure 2:**
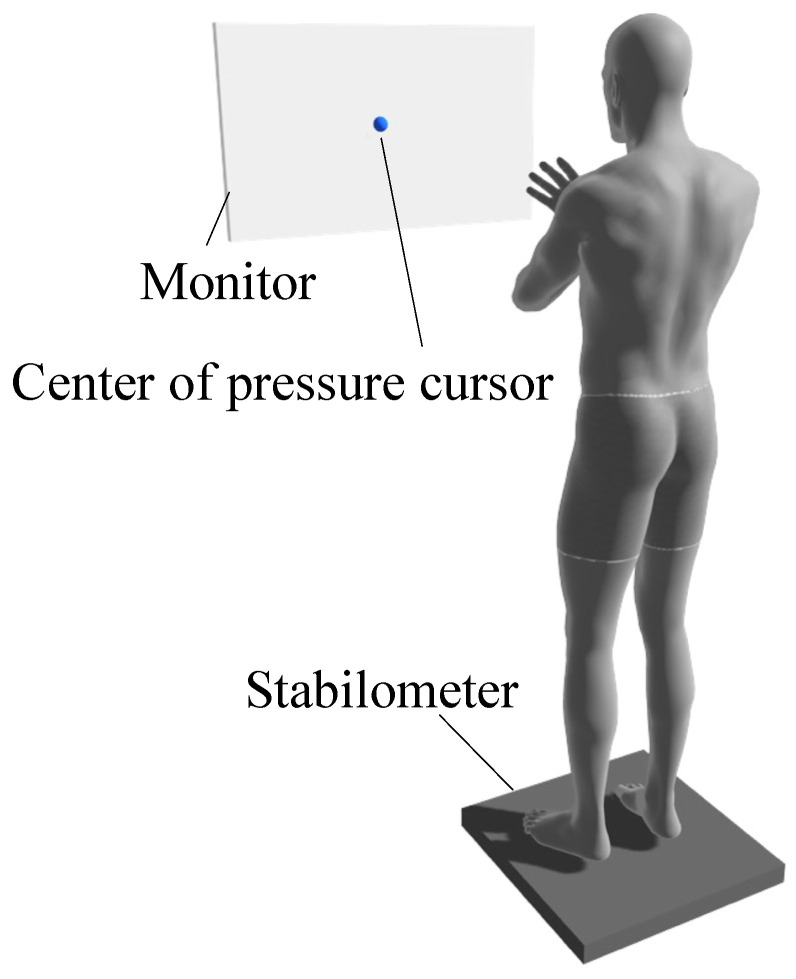
The participants performed a standing postural control task by standing on foam rubber placed on a stabilometer with both arms folded in front of their chest. The participants wore an electroencephalogram on their heads. A monitor was placed in front of each participant, which displayed only the center of the pressure cursor at that time.

**Figure 3:**
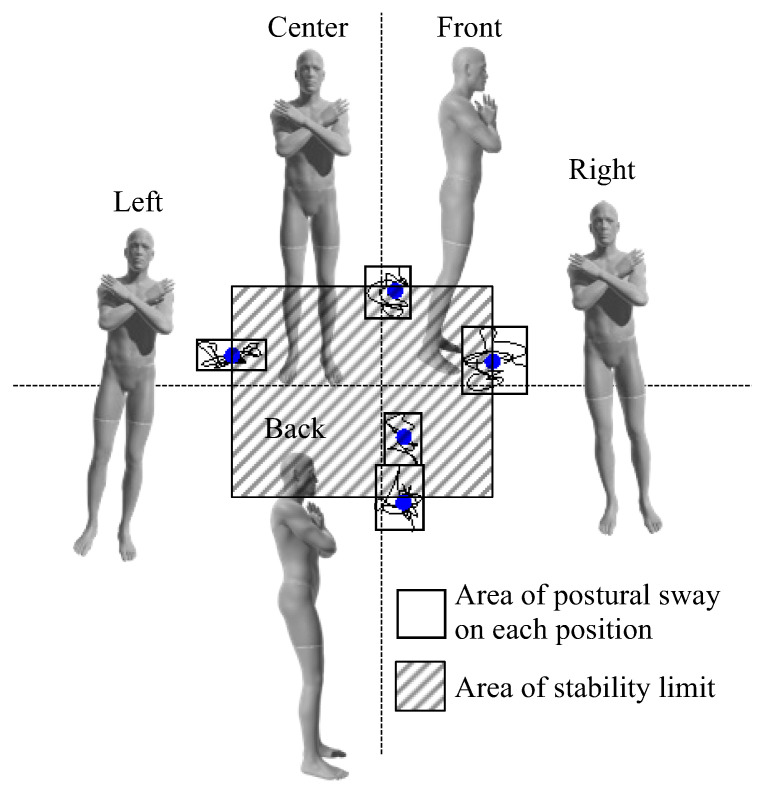
The IPS was determined for all participants. The center of pressure sway, for 10 s, was measured for each participant in the center and in different postures including tilted front, back, right, and left maximally. The gray area indicates the area of postural sway on each position. The shaded area indicates the area of stability limit. IPS: index of postural stability.

Foam rubber (ANIMA Co., Ltd., Tokyo, Japan) was placed on a stabilometer (T.K.K. 5810; Takei Kiki Kogyo Co., Ltd., Niigata, Japan). The participants stood barefoot on the stabilometer with the inside of their feet 10 cm apart. A monitor was placed in front of the participant at eye level. The monitor displayed the center of a pressure cursor at that time (Figure 2). The postural control task was used to determine the index of postural stability (IPS) [28], which has been used to assess age-dependent changes in balance ability [28] and balance control in a wide range of subjects, including healthy middle-aged and older adults [29], pregnant women [30], and children with cerebral palsy [31]. The IPS has also been used to evaluate athletes, as it shows no ceiling effect on healthy young subjects [28]. After 10 s of postural sway measurement in the center of the base plane of the support, pastural sway was measured for 10 s with the center of pressure shifted maximally to the front, back, right, and left (Figure 3). The sequence for all measurements was anterior, posterior, right, and left. During the measurement, the participants were instructed to suppress the center of gravity sway as much as possible, maintain an upright posture, and perform the measurement with all plantar surfaces connected to the ground.

The verbal instructions for the “no attentional instruction”, IF, and EF conditions were as follows. During the “no attentional instruction condition”, the focus of attention was not referred to during the measurement and the participants were instructed to “lean as far as possible and hold it for 10 s.” In the IF condition, the participants were instructed to focus their attention on their feet and keep their weight in front (back, right, left) of their feet for 10 s. This was done to focus their attention on the inside of the body. In the EF condition, we instructed the participants to focus their attention on the cursor on the monitor and move the cursor as up (down, right, left) as possible from the center and hold it for 10 s [32]. This allowed for focus on the outside of the body.

Immediately after the attention focus, the subjective percentage rating was assessed, which is based on the numerical rating scale (0–100). The participants were asked to self-evaluate their attention on the inside and outside of the body, as instructed. Those who scored <60 on the subjective percentage rating were excluded from the study because of insufficient attention focus [33].

### 2.3. Measures

The IPS was calculated as “IPS = log [(area of stability limit + area of postural sway)/area of postural sway]” from stabilometer data [28]. The area of postural stability was calculated by averaging the postural sway areas in five positions, and the area of stability limit was demined by the front and rear center movement distance between the front and back positions × the distance between right and left positions (Figure 3). The participants whose IPS in the IF condition was higher than that in the EF condition were referred to as the IF-dominant group and those whose IPS in the EF condition was higher than that in the IF condition were referred to as the EF-dominant group, as previously described [21].

Polymate Pro (MP-6100, Miyuki Giken Co., Ltd., Tokyo, Japan) and active dry electrodes (Miyuki Giken, Co., Ltd., Tokyo, Japan) were used to measure the EEG signals. The earth electrodes were placed on the left earlobe. An external input cable was used to connect the stabilometer to the electroencephalogram and synchronized the trigger to start recording. In addition, EEG was recorded in 19 channels (Fp1, Fp2, F7, F3, Fz, F4, F8, T3, C3, Cz, C4, T4, T5, P3, Pz, P4, T6, O1, and O2) according to the international 10–20 system. A reference electrode was placed in the left ear lobe. The sampling rate was 1000 Hz. The theta frequency band (6.5–8 Hz) was used during monitoring as it is a measure of attention and cognition [34,35]. In addition, previous reports showed that activity in the theta-wave band was more sensitive to cognitive phases in young adults [36], and high theta-wave activity in postural control tasks was associated with high attentional demands and error detection [37]. Therefore, in this study, the analysis of the theta-wave band may provide results on the dominance of the attention focus in standing postural control, rather than EEG activity during postural control alone.

### 2.4. Data Analysis

First, the Shapiro–Wilk test was used to examine the normality of the data. Next, participant characteristics and IPS in the IF and EF conditions were compared between the groups using an unpaired *t*-test. Statistical analyses were performed using SPSS ver. 24.0 (IBM, Chicago, IL, USA).

Cortical activity in the IF and EF conditions was compared between the IF-dominant and EF-dominant groups. The recorded EEG data were downsampled to 512 Hz using EEGLAB in MATLAB (Mathworks, Inc., Natick, MA, USA), and the bandpass filter was set at 1–40 Hz. Independent component analysis was then performed to remove limiting factors such as blinking, heartbeat, muscle activity, and channel noise. The EEG data was then divided into epochs of 1 s each. Next, exact low-resolution brain electromagnetic tomography (eLORETA) was used to reconstruct the cortical current density distribution from the normalized EEG data. eLORETA analysis was performed using the Montreal Neurological Institute (MNI) 152 template. In eLORETA, the coordinates of 19 electrodes were first included in a probabilistic anatomical template of the Talairach atlas. These coordinates were then used to compute the eLORETA transformation matrix. After conversion to an average reference EEG activity, 1 s epochs, without limiting factors, were averaged, and cross-spectra were calculated in eLORETA for each participant’s theta-wave frequency band. The eLORETA transformation matrix was then used to convert the theta frequency band cross spectra into eLORETA files. Based on the eLORETA log-transformed current power [38], corresponding F-tests were performed for each voxel in the theta-wave band. From the 3D images obtained by statistical analysis, voxels showing significant differences were detected by statistical non-parametric mapping. The statistical significance level was set at 0.05.

## 3. Results

Age, height, and weight were compared between the IF- and EF-dominant groups and no significant differences were observed (*p* > 0.05, Table 1).

**Figure 4:**
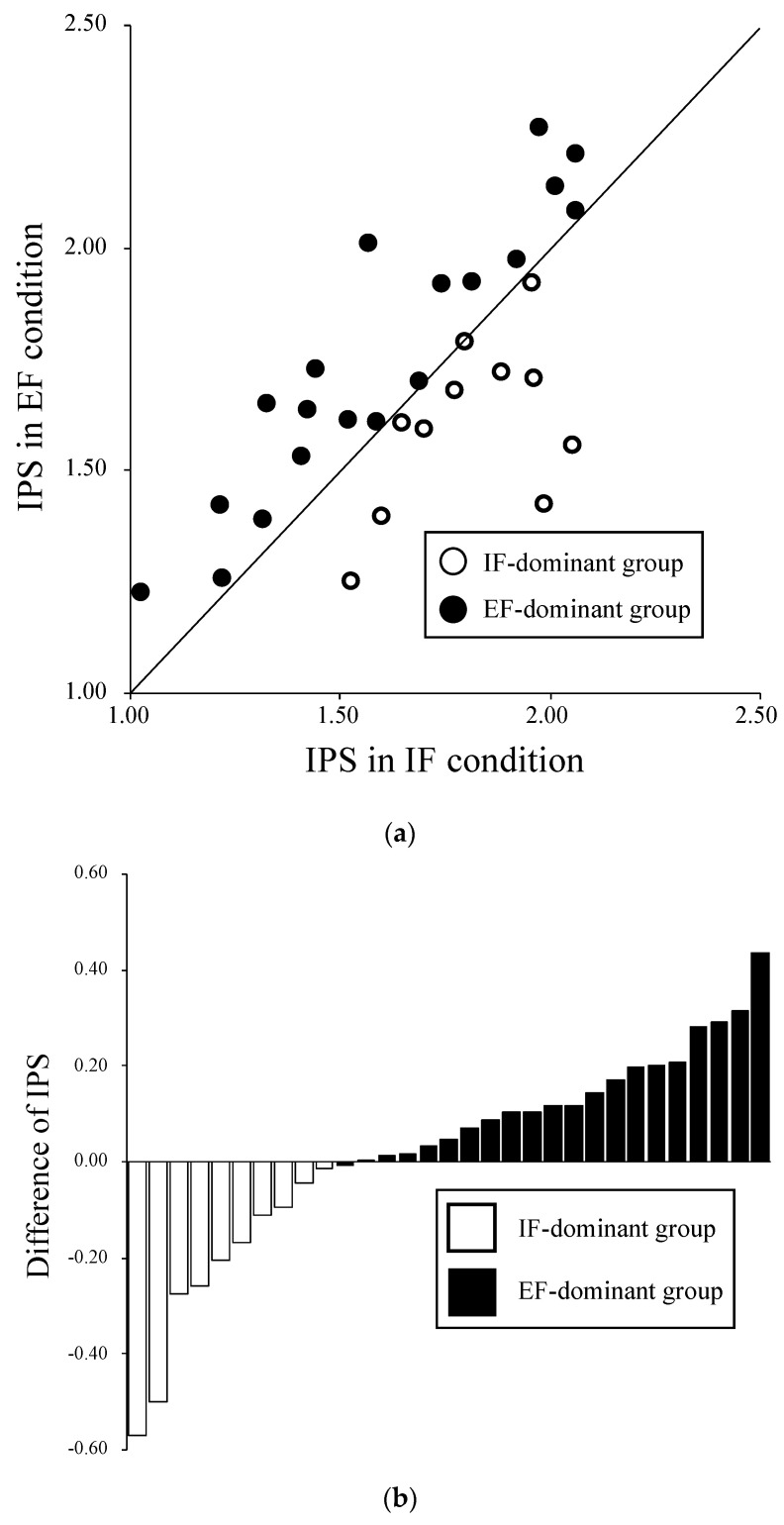
(**a**) The scatter plot depicts the IPS in the IF and EF conditions. Participants whose IPS in the EF condition was higher than that in the IF condition were classified as the IF-dominant group. Those whose IPS in the EF condition was higher than that in the IF condition were classified as the EF-dominant group. (**b**) The EF-dominant group included participants with a positive difference in IPS. By contrast, participants with a negative difference of IPS were included in the IF-dominant group. EF: external focus; IF: internal focus; IPS: index of postural stability.

The participants were classified into two groups as follows: the IF-dominant group (*n* = 11) and the EF-dominant group (*n* = 20), based on previous studies [21]. The IF-dominant group consisted of participants whose IPS was higher in the IF condition than in the EF condition. Conversely, the EF-dominant group consisted of participants whose IPS was higher in the EF condition than in the IF condition (Figure 4a). The IPS in the IF condition was significantly higher in the IF-dominant group than that in the EF-dominant group (*p* = 0.02, Figure 4b). By contrast, IPS in the EF condition was higher in the EF-dominant group than in the IF-dominant group (*p* = 0.05, Figure 4b), although no significant difference was identified between the groups.

**Figure 5:**
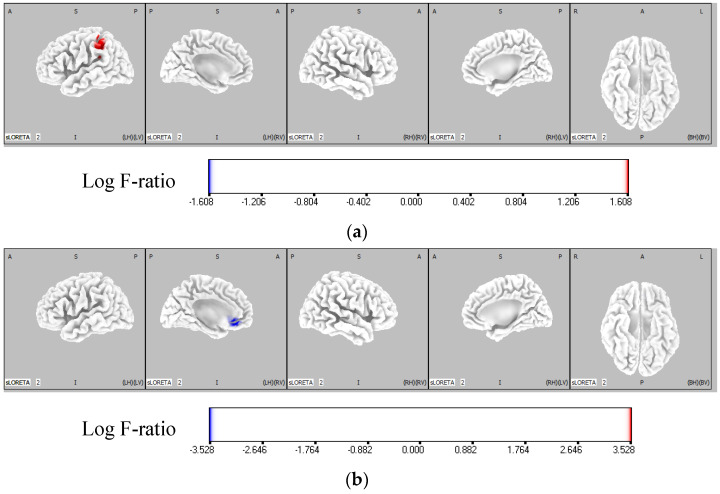
Comparison of theta activity of each group in IF and EF conditions. The red region represents the area where the theta activity was significantly higher in the IF-dominant group than the EF-dominant group. The blue region shows the area where the theta activity is significantly higher in the EF-dominant group than the IF-dominant group. The color bar represents the voxel log F-ratio values. (**a**) Comparison of the theta activity between the IF-dominant and EF-dominant groups in the IF condition. In the IF condition, the activity of the left parietal lobe (BA40) was significantly higher in the IF-dominant group than in the EF-dominant group (*p* < 0.05). (**b**) Comparison of the theta activity between the IF-dominant and EF-dominant groups in the EF condition. In the EF condition, the activity of the left frontal lobe (BA32) was significantly higher in the EF-dominant group than in the IF-dominant group (*p* < 0.05). EF: external focus; IF: internal focus.

The results of the eLORETA analysis revealed that in the IF condition, theta activity was significantly higher in the left parietal lobe (BA40) of the IF-dominant group compared to the EF-dominant group (*p* < 0.05). In the EF condition, theta activity was significantly higher in the left frontal lobe (BA32) of the EF-dominant group than that in the IF-dominant group (*p* < 0.05) (Figure 5, Table 2).

## 4. Discussion

In this study, the dominance of attention focus in standing postural control was examined using a stabilometer. The results showed that the IF-dominant group (*n* = 11) performed better in the IF condition and the EF-dominant group (*n* = 20) performed better in the EF condition; Sakurada et al. [20,21,22] reported that dominance of attention focus was found in the upper limb tracking task. The differences between the IF-dominant and EF-dominant groups included their motor imagery ability [20], brain activity [21], and sensory information processing [22]. The results of this study showed that the dominance of attention focus, observed in the upper limb tracking task, was also present in the standing postural control task.

In our investigation of the neural basis for the dominance of attention focus in standing postural control by using EEG, higher theta activity was observed in the left parietal lobe of the IF-dominant group than the EF-dominant group in the IF condition. Dominguez et al. [39] reported an increase in theta activity in the parietal lobe during a postural control task that relied on proprioceptive and vestibular sensory stimuli. Furthermore, Reichenbach [40] reported that superficial and proprioceptive processing were associated with theta activity in the parietal region using transcranial magnetic stimulation. Here, the participants focused on their feet in the IF condition, which may have promoted superficial and proprioceptive-dominated postural control from the feet. Therefore, the IF-dominant group may have performed the task with sensory-dominant postural control, as additional sensory processing occurred in the IF condition compared to the EF-dominant group. Therefore, it is possible that the IF-dominant group experienced additional sensory processing in the IF condition than the EF-dominant group and performed the task with sensory-dominant postural control. This interpretation is also consistent with the view of Sakurada et al. [22], who reported that the IF-dominant group prioritized somatosensory processing. Huizeling et al. [41] reported that elderly people with difficulty maintaining concentration showed decreased parietal lobe theta activity during cognitive tasks, suggesting that parietal lobe theta activity reflects attentional control. Additionally, Ellmers et al. [42] reported that attentional focus on the IF causes conscious control of the action, and thus a high level of attentional control is required in the IF condition. The IF-dominant group had a higher attentional control ability and may have improved their performance in the IF condition, where conscious control is required.

By contrast, significantly higher theta activity was observed in the left frontal lobe of the EF-dominant group than that of the IF-dominant group in the EF condition. Previous studies [35,43,44,45] have reported that frontal theta activity increases during a cognitive task, suggesting that the participant is concentrating on the task, and that this is associated with cognitive control [46]. Furthermore, Dominguez et al. [39] identified a strong correlation between frontal theta activity and postural sway in a standing postural control task under open-eyed conditions; higher theta activity was detected, and less postural sway was observed. These results suggest that the EF-dominant group may have focused selectively on the center of pressure cursor in the EF condition compared with the IF-dominant group. Several previous studies [47,48,49] have reported that frontal theta activity is associated with error detection in standing postural control tasks. Furthermore, Sherman et al. [19] reported that, in a postural control task with a one-legged stance, a decrease in postural sway and an increase in frontal theta activity occurred in the EF condition compared with the IF condition, and that error detection based on visual feedback contributed to the results in the EF condition. In the EF condition in this study, attention was focused on the COP cursor on the monitor, and error detection should be based on visual feedback, as in previous studies. The results suggest that the EF-dominant group, which showed improved performance in this environment, tended to perform added detailed error detections.

This study had some limitations. First, whether the participants were able to accurately focus their attention on the inside and outside of their bodies was unclear. Here, a subjective percentage rating was employed based on previous studies, although it could not be examined as it was a self-assessment of the participants, and no objective measure of the accuracy of attentional focus was implemented. Second, the EEG recorded in this study had 19 channels. Although the validity and reliability of the measurement results of the international 10–20 method have been verified [50,51], the reliability of the recorded data is said to improve as the number of EEG channels increases [52]. Therefore, compared to studies using numerous channels, this study may have less power. Third, we only analyzed the theta-wave frequency band in this study. Future studies should examine EEG activity in other frequency bands. Fourth, this study only examined performance and not learning effects. Thus, conducting a longitudinal study in the future might be necessary to examine not only performance, but also the learning effect and the training effect using IF or EF.

## 5. Conclusions

In this study, the IF-dominant group showed a higher parietal activity related to somatosensory processing and attentional control, while the EF-dominant group showed a higher frontal activity related to cognitive control and error detection. This supports the dominance of attention focus and indicates that verbal instruction that takes the attention focus into account may maximize performance in standing postural control. This study thus contributes to the development of training methods that consider the dominance of attention focus in standing postural control.

## Figures and Tables

**Table 1:** Characteristics of the IF-dominant group (*n* = 11) and EF-dominant group (*n* = 20).

	IF-Dominant Group	EF-Dominant Group	*p*-Value
	Mean ± SD	Mean ± SD	
Age (years)	21.18 ± 0.60	21.00 ± 0.79	0.28
Height (cm)	172.91 ± 4.87	172.35 ± 5.82	0.39
Body weight (kg)	68.36 ± 10.70	64.50 ± 5.97	0.10

EF: external focus; IF: internal focus.

**Table 2:** Brain regions with significantly higher activity between the IF-dominant and EF-dominant groups.

Condition	Group	Brain Region	BA	MNI Coordinates	*p*-Value
				(x, y, z)	
IF	IF > EF	Left inferior parietal lobule cortex	40	−40, −40, 45	*p* < 0.05
EF	EF > IF	Left anterior cingulate cortex	32	−5, 35, −5	*p* < 0.05

BA: Brodmann area; EF: external focus; IF: internal focus; MNI: Montreal Neurological Institute.

## Data Availability

The data that support the findings of this study are available on request from the corresponding author. The data are not publicly available because they contain information that can compromise the privacy of research participants.
